# Upregulation of homeobox D10 expression suppresses invasion and migration of clear cell renal cell carcinoma through targeting of E-cadherin

**DOI:** 10.1007/s11033-021-06993-8

**Published:** 2021-11-25

**Authors:** Zongtao Ren, Yunfeng Niu, Bo Fan, Aili Zhang

**Affiliations:** 1 Department of Urology, The Fourth Hospital of Hebei Medical University, No. 12 Jian-Kang Road, Shijiazhuang, 050011 Hebei Province China; 2 Laboratory of Pathology, Hebei Cancer Institute, The Fourth Hospital of Hebei Medical University, Shijiazhuang, China

**Keywords:** CCRCC, HOXD10, E-cadherin, Invasion

## Abstract

**Background:**

Clear cell renal cell carcinoma (CCRCC) is one of the most common types of renal cell carcinoma. Accumulating evidence indicates that homeobox D10 (HOXD10) acts as a tumor suppressor or oncogene in various carcinomas. However, the regulation and potential mechanisms of HOXD10 in CCRCC remain largely unknown.

**Purpose:**

To explore the effect and potential mechanism of HOXD10 on the invasion and migration of CCRCC cells.

**Methods:**

The expression of HOXD10, E-cadherin and other epithelial mesenchymal transition (EMT)-related proteins was assessed by reverse transcription-quantitative real-time PCR (qRT-PCR) and Western blots. A series of functional assays were performed in RCC cell lines to explore the function of HOXD10 in CCRCC progression. Bioinformatics analysis, ChIP assays, and dual luciferase reporter assays were utilized to identify the interaction between HOXD10 and E-cadherin.

**Results:**

Low expression of HOXD10 and E-cadherin was observed in CCRCC tissues and ACHN and 786-O cells. Downregulation of HOXD10 expression was correlated with the TNM stage of CCRCC patients. Functional experiments demonstrated that malignant biological ability was significantly inhibited by HOXD10 overexpression in RCC cells. Moreover, E-cadherin was a potential target gene of HOXD10, as evidenced by a series of assays. In addition, overexpression of HOXD10 inhibited the progression of CCRCC by regulating the expression of E-cadherin, vimentin, and β-catenin in vitro.

**Conclusion:**

HOXD10 acts as a tumor suppressor and suppresses invasion and migration of CCRCC cells by regulating E-cadherin and EMT processes. Thus, targeting HOXD10 may be a therapeutic strategy for CCRCC treatment.

**Supplementary Information:**

The online version contains supplementary material available at 10.1007/s11033-021-06993-8.

## Introduction

Renal cell carcinoma (RCC) is one of the most common tumors of the kidney and arises from the epithelium of the renal tubules [1]. Although RCC can be completely cured surgically in the early stages, approximately 30% of RCC tumors are already metastatic at the initial diagnosis, while 30–40% of patients develop local recurrence or distant metastasis after the initial treatment [2]. RCC is not sensitive to traditional radiotherapy and chemotherapy. The most common histological subtype of RCC is clear cell renal cell carcinoma (CCRCC), which accounts for approximately 75% of all cases, followed by papillary RCC (15%) and chromophobe RCC (5%) [3]. Patients with metastatic CCRCC have a worse prognosis than those with non-CCRCC. Given the poor prognosis of CCRCC, elucidation of the precise molecular mechanisms of tumorigenesis, invasion and metastasis of CCRCC and identification of a novel biomarker related to CCRCC are urgently needed.

Homeobox (HOX) genes were first discovered in *Drosophila melanogaster* [4]. The human genome contains a highly conserved homeobox that can encode a highly conserved family of transcription factors. HOX transcription factors can bind to specific promoters on DNA. Accumulating studies indicate that dysfunction of HOX transcription factors plays key roles in the progression of various types of carcinomas [5]. Homeobox genes are classified as HOX-I and HOX-II. In humans, HOX genes belong to HOX-I, which clusters into 4 groups (HOXA, HOXB, HOXC, HOXD), and each gene is named 1 to 13. HOX genes regulate the expression of genes involved in cell–cell and cell-extracellular matrix interactions [6, 7]. As an important member of the HOX gene family, homeobox D10 (HOXD10) plays an important role in carcinogenesis by regulating cell invasion, migration, and proliferation. HOXD10 is an inhibitor of tumor metastasis [5], and its expression is significantly downregulated in several types of carcinomas, such as prostate cancer, breast cancer, and liver cancer [8–10]. For example, previous experiments demonstrated that the expression of HOXD10 was prominently decreased in HCC tissues, and overexpression of HOXD10 could inhibit cell invasion and migration [8]. This result indicated the important role of HOXD10 in tumorigenesis as an inhibitor of tumor metastasis. However, the clinical relevance, biological function, and precise molecular mechanism of HOXD10 in CCRCC remain unclear.

Previous studies have demonstrated that most HOX transcription factors regulate the invasion and migration of tumor cells by recruiting downstream targets to initiate EMT. In the course of tumor progression, EMT is the basis for invasion and metastasis of malignant cells, and loss of E-cadherin leading to the destruction of cell–cell contacts is considered an essential event in EMT [11].

In the present study, the expression of HOXD10 was analyzed in CCRCC tissues, corresponding normal renal tissues, and RCC cell lines. Through a series of cellular assays, we detected the effects of HOXD10 overexpression and knockdown on RCC cell biological behavior. Finally, we proved that HOXD10 suppresses invasion and migration through upregulation of the expression of a potential downstream gene, E-cadherin.

## Materials and methods

### Patients and specimens

A total of 72 CCRCC tissues paired with corresponding normal tissues were obtained from the Department of Urology, the Fourth Hospital of Hebei Medical University, between 2017 and 2019. All histological specimens were diagnosed as CCRCC by senior pathologists. Tissue samples were frozen in liquid nitrogen immediately after the operation. One part of these tissue samples was fixed with formalin, and the remaining part was frozen at − 80 °C to extract RNA. The TNM and nuclear grades were determined according to the 2009 TNM classification system and the Fuhrman nuclear grading system. The clinicopathological and clinical data in the study cohort are summarized in Table 1.

**Table 1:** HOXD10 mRNA levels in relation to clinical-pathological parameters of CCRCC patients

Group	N	Mean ± SD	t	*P*-value
Age (years)				
≤ 60	49	0.424 + 0.534	− 0.351	0.727
> 60	23	0.484 + 0.914		
Gender				
Male	49	0.384 + 0.581	− 1.092	0.279
Female	23	0.569 + 0.835		
Lymph node metastasis				
Negative	66	0.473 + 0.695	1.245	0.217
Positive	6	0.117 + 0.074		
Histological grade				
Well/moderate	65	0.464 + 0.694	0.823	0.413
Poor	7	0.244 + 0.370		
TNM stage				
I	49	0.493 + 0.537	2.963	0.038
II	8	0.479 + 0.647		
III	6	0.079 + 0.067		
IV	9	0.049 + 0.023		

*SD* standard deviation, *t* Student’s t test

### Cell culture and transfection

The human RCC cell lines 786-O (RRID: CVCL_1051) and ACHN (RRID: CVCL_1067) and human embryonic kidney 293 T cells (RRID: CVCL_0063) were purchased from the National Collection of Authenticated Cell Cultures (Shanghai, China). The cell lines were cultured in RPMI-1640 medium (Gibco, USA) supplemented with 10% fetal bovine serum (FBS) (Invitrogen, USA), 100 U/ml penicillin, and 100 μg/ml streptomycin. The cell lines were incubated in a humidified atmosphere of 5% CO_2_ at 37 °C. When the cell confluence reached 80%, the cells were subcultured at a ratio of 1:3.

The overexpression plasmid pcDNA3.1-HOXD10 (RRID: Addgene_17467) was synthesized by GenePharma (Shanghai, China). Four short hairpin RNAs (shRNAs) targeting HOXD10 were provided (sh-HOXD10-1/2/3/4, RRID: Addgene_31971, GenePharma, China) to avoid off-target effects. The relevant vector (pcDNA3.1-NC) and negative control shRNA (sh-NC) were used as negative controls. Cells were seeded into a 6-well plate (2 × 10^5^ cells/well). ACHN/786-O cells were transfected with pcDNA3.1-HOXD10 or sh-HOXD10.

### Quantitative real-time PCR (qRT-PCR)

Total RNA was extracted from tissues and cell lines by TRIzol reagent (Invitrogen, China). cDNA was synthesized using the Transcriptor First-Strand cDNA Synthesis Kit (Roche, Switzerland) according to the manufacturer’s instructions. Quantitative real-time PCR (qRT-PCR) was performed with GoTaq qPCR Master Mix (Promega, USA) and an Applied Biosystems 7500 Detection system (Applied Biosystems, USA). The human GAPDH gene was used as an internal control. The 2^−△△CT^ method was used to calculate the relative expression of target genes [12]. The primer sequences are listed in Supplementary Table S1.

### Western blot analysis

Total proteins were extracted from transfected cells using RIPA lysis buffer containing PMSF (Solarbio) and protease inhibitor cocktail (Promega). The protein concentration was determined by a BCA Protein Assay Kit (Multi Sciences). After the samples were mixed with loading buffer and heated at 99 °C for 5 min, the protein lysates were separated by 12% SDS and transferred onto PVDF membranes (Millipore). The transferred membranes were blocked with 5% skim milk for 1 h at room temperature and incubated overnight with specific primary antibodies at 4 °C. Subsequently, the membranes were incubated at room temperature with horseradish peroxidase–conjugated goat anti-rabbit IgG (KPL) for 1 h. The bands were visualized with enhanced chemiluminescence (ECL) detection reagent (Multi Sciences) by Chemi XT 4 (Syngene). The primary antibodies used were anti-HOXD10 (RRID: AB_2049745), anti-E-cadherin (RRID: AB_2660958), anti-vimentin (RRID: AB_2492284), anti-β-catenin (RRID: AB_2811667), and anti-β-actin (RRID: AB_1873358).

### Immunohistochemistry (IHC)

HOXD10 protein expression was detected by immunostaining, which was performed on paraffin-embedded tumor tissue sections (4 μm thick) and corresponding normal tissue sections (4 μm thick). Paraffin sections without antibody were used as negative controls. A rabbit anti-human polyclonal antibody against HOXD10 (1:200 dilution) was used to detect the protein expression of HOXD10. The scores of the staining intensity and range were used as the index of the result analysis. All of the slides were examined blindly by three experienced pathologists.

### Cell invasion assay

Cell invasion assays were evaluated in 24-well Transwell chambers (Corning, USA). The Transwell chambers were coated with 30 µg Matrigel and incubated at 37 °C for 2 h. The upper chambers were supplemented with 5 × 10^5^ cells/well, and RPMI-1640 medium containing 10% FBS was added to the lower chambers. After 24 h at 37 °C, the cells were fixed with paraformaldehyde (4%) and stained with crystal violet. The cell numbers were counted in five random fields (at × 100 magnification) per filter under a microscope. The assays were repeated in triplicate.

### Wound-healing assay

Cells were seeded in 6-well plates. When the ratio of adherent cells reached 90%, the serum was removed to starve cells for 24 h, and then, a wound was scratched with a 200 µL pipette tip in the cultured cells. The images were captured at the same position of each well 0 h, 12 h, and 24 h after scratching under a microscope. The relative distance of cell migration to the scratched area was measured, and the healing percentage was calculated. The assays were repeated in triplicate.

### Colony formation assay

Approximately 2000 cells were seeded on six-well plates and incubated for 14 days. The cells were fixed with 4% paraformaldehyde and stained with 0.5% crystal violet, and then, the cell clusters in each well were counted. All assays were repeated in triplicate.

### MTS assay

The cells were seeded in 96-well plates (1 × 10^3^ cells/well). At 0, 24, 48, 72, and 96 h after seeding, 20 ml MTS (Promega, USA) was added to each well at the same time of every day and then coincubated for 2 h before absorbance measurement. All assays were performed in triplicates.

### Chromatin immunoprecipitation (ChIP) assay

The ChIP assay was performed according to EZ-Magna ChIP A/G (Millipore, USA) instructions. The lysate was immunoprecipitated with a rabbit IgG or HOXD10 antibody, and the ChIP-derived DNA was amplified using qRT-PCR. The binding primer sequences were located in the promoter region of E-cadherin: forward: 5′- CGAGAGCTACACGTTCACGG-3′, reverse: 5′- GGCCTTTTGACTGTAATC ACACC-3′.

### Luciferase reporter assay

pmirGLO-E-cadherin (WT) (RRID: Addgene_68363) and pmirGLO-E-cadherin (MUT) were cotransfected with HOXD10 mimics or HOXD10-NC into ACHN cells using Lipofectamine 2000. Luciferase activity was measured with a Dual-Luciferase Reporter Assay System (Promega) at 48 h after transfection and normalized to Renilla luciferase activity.

### Statistical analysis

Statistical analysis was performed using SPSS 21.0 (IBM Company, USA) and graphed using GraphPad Prism 5.0. The data are expressed as the mean ± SD, and the means were compared using the chi-squared test and Student’s t-test. Bivariate correlations between study variables in tissues were calculated by Spearman correlation analysis. Two-sided tests were used to determine significance, and *P* < 0.05 was regarded as statistically significant.

## Results

### HOXD10 expression was downregulated in CCRCC tissues and RCC cell lines

**Fig. 1:**
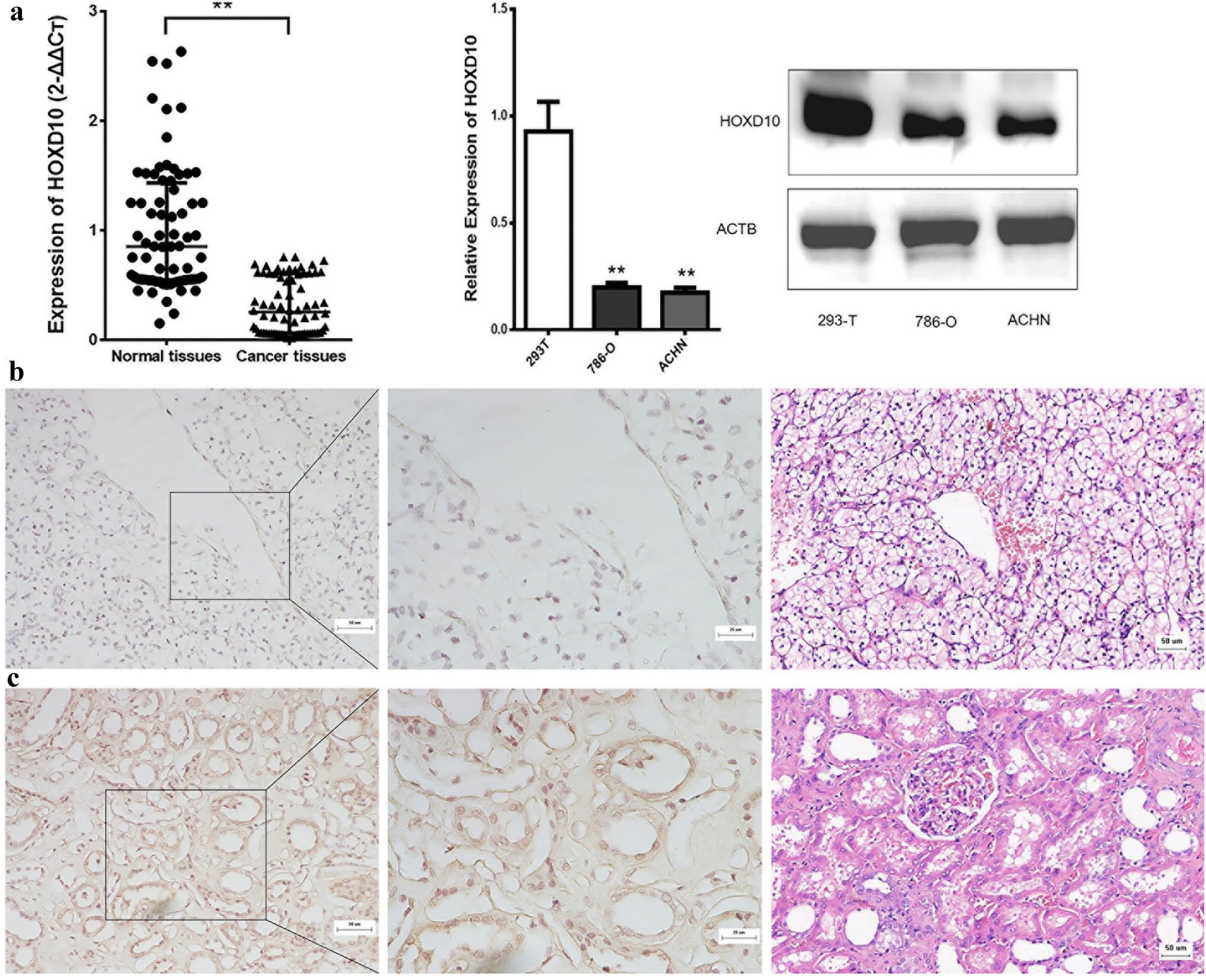
HOXD10 expression was downregulated in CCRCC tissues. **a** HOXD10 expression was significantly downregulated in CCRCC tissues compared to normal renal tissues. mRNA and protein levels of HOXD10 in 786-O, ACHN and 293-T cells, **P < 0.01. **b** Negative immunohistochemical and HE staining of HOXD10 in CCRCC tissues (SP × 200, SP × 400). **c** Positive immunohistochemical and HE staining of HOXD10 in normal renal tissues (SP × 200, SP × 400)

To verify whether HOXD10 plays a role in the occurrence and progression of CCRCC, we used the StarBase-V3.0 database to demonstrate that the expression level of HOXD10 was downregulated in CCRCC tissues compared with the corresponding normal renal tissues (Supplementary Fig. S1). Then, the mRNA and protein levels of HOXD10 in 72 paired CCRCC tissues were detected by qRT-PCR and IHC. The mRNA levels of HOXD10 were remarkably downregulated in the CCRCC tissues compared with the normal renal tissues (*P* < 0.01, Fig. 1a). IHC staining indicated that HOXD10 protein was mainly expressed in the nucleus. However, the positive staining intensity in the CCRCC tissues was significantly attenuated compared with that in the normal renal tissues (Fig. 1b, c). To verify the above results, we detected the mRNA and protein levels in the cell lines 786-O, ACHN and 293 T. The results showed that the expression of HOXD10 was significantly downregulated in the RCC cell lines compared with the 293 T cell line at both the mRNA and protein levels (Fig. 1a). Next, we analyzed the relationship between HOXD10 and clinicopathologic features. Notably, the expression levels of HOXD10 were related to clinical stage (*P* < 0.05) but not to age, sex, lymph node metastasis, or pathological differentiation (*P* > 0.05) (Table 1).

### Overexpression of HOXD10 inhibited the invasion, migration, proliferation, and colony formation of RCC cells

**Fig. 2:**
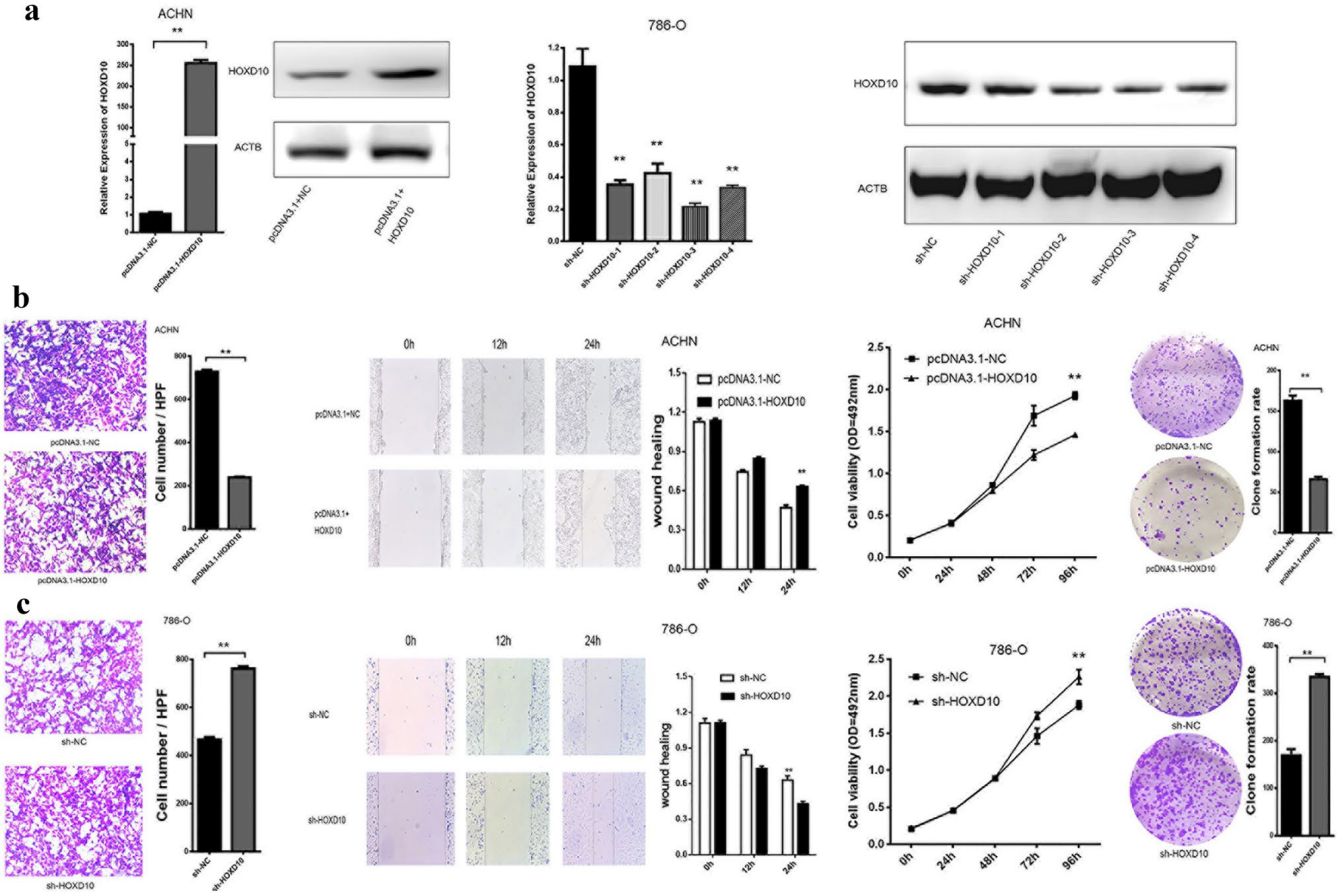
Functional analysis of HOXD10 in human RCC cell lines. **a** The expression level of HOXD10 in RCC cells transfected with pcDNA3.1- HOXD10 or four sh-HOXD10 sequences compared with the negative control (NC), **P < 0.01. **b** Transwell invasion assays showed that overexpression of HOXD10 inhibited the invasive ability of ACHN cells. **P < 0.01. Wound healing experiments showed that overexpression of HOXD10 inhibited the migration of ACHN cells. **P < 0.05. MTS assays showed that overexpression of HOXD10 reduced the proliferation of ACHN cells. **P < 0.05. Overexpression of HOXD10 reduced the colony formation of ACHN cells. **P < 0.01. **c** Knockdown of HOXD10 enhanced invasion, migration, proliferation, and colony formation of the 786-O cell line

We investigated the biological function of HOXD10 in RCC cells. Plasmid-mediated overexpression and shRNA-mediated knockdown were established in ACHN and 786-O cell lines, respectively (Fig. 2a). By comparing the expression of the four groups of shRNA-mediated knockdown cells, we found that the expression level of HOXD10 in the sh-HOXD10-3 group was the lowest. Therefore, we selected the sh-HOXD10-3 group for subsequent experiments. The invasion and migration of ACHN cells was dramatically inhibited by HOXD10 overexpression (*P* < 0.05, Fig. 2b). The MTS assay showed that upregulation of HOXD10 expression significantly suppressed the proliferation of ACHN cells compared with control cells (*P* < 0.05, Fig. 2b). Moreover, fewer colonies were formed by the ACHN cells transfected with pcDNA3.1-HOXD10 than the control cells (*P* < 0.01, Fig. 2b). In contrast, knockdown of HOXD10 significantly promoted invasion and migration of 786-O cells (*P* < 0.05, Fig. 2c), and knockdown of HOXD10 markedly inhibited proliferation and clonogenic survival of 786-O cells (*P* < 0.05, Fig. 2c).

### E-cadherin was a direct target of HOXD10

**Fig. 3:**
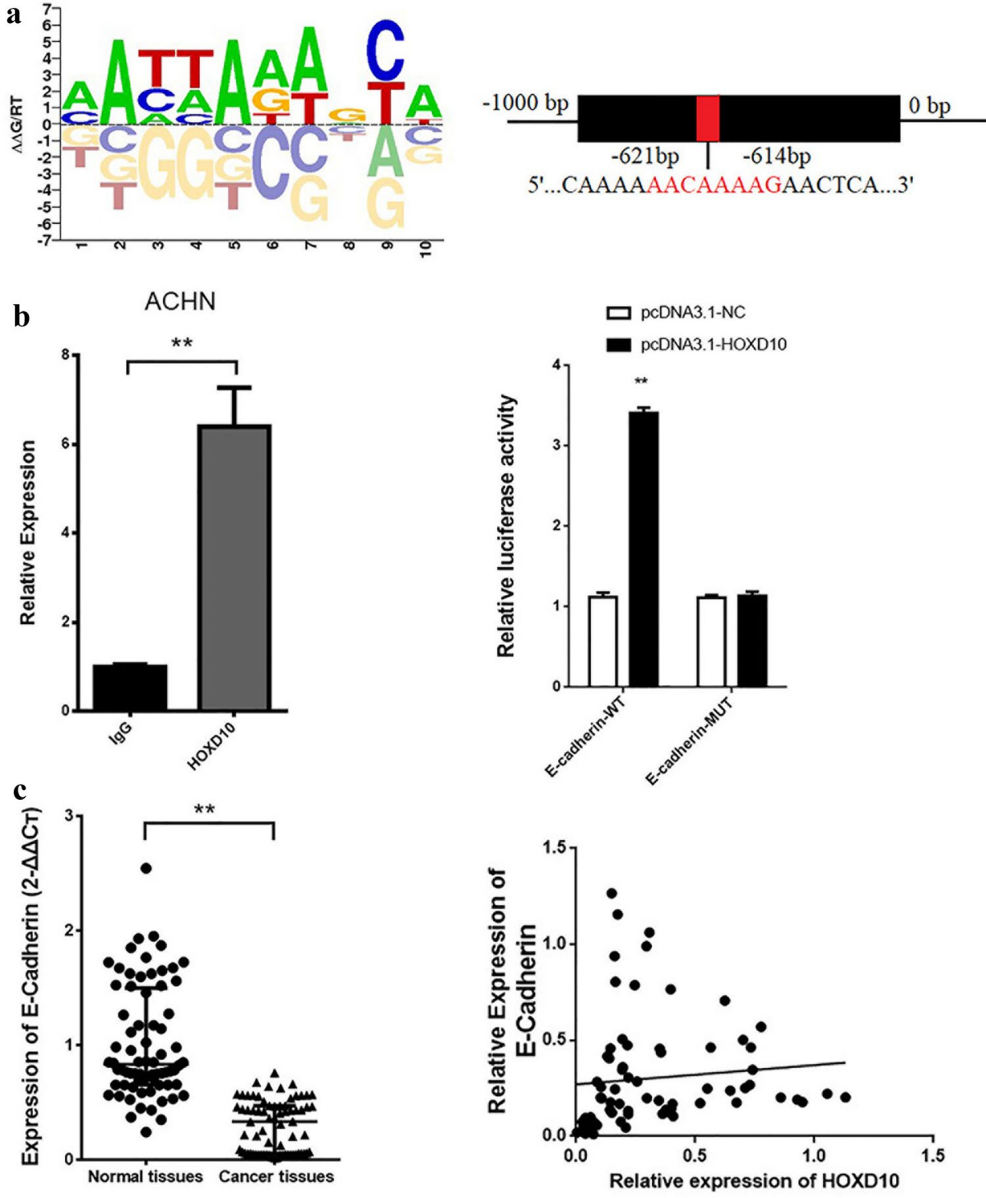
E-cadherin is a potential target gene of HOXD10 and its expression is downregulated in CCRCC tissues.** a** Prediction of HOXD10 binding sites in the E-cadherin promoter region. **b** ChIP assays showed that HOXD10 could bind to the promoter region of E-cadherin. **P < 0.01. Validation of the interaction between HOXD10 and the E-cadherin promotor region by dual luciferase reporter assays. **P < 0.01. **c** E-cadherin expression was significantly downregulated in CCRCC tissues compared to normal renal tissues, **P < 0.01. The correlation between HOXD10 and E-cadherin expression was analyzed in CCRCC tissues, Rs = 0.454, *P* < 0.01

To investigate the potential target of HOXD10, we searched the PROMO website. According to the prediction, the E-cadherin 3′-UTR had a potential binding site for the HOXD10 sequence (Fig. 3a). The ChIP assay showed that HOXD10 bound to the E-cadherin upstream sequence (*P* < 0.01, Fig. 3b). In addition, a dual luciferase reporter assay further revealed that luciferase activity was prominently enhanced by pcDNA3.1-HOXD10 with the E-cadherin-WT vector compared with that of the control group, but there was no obvious difference in the E-cadherin-MUT group regardless of transfection with pcDNA3.1-HOXD10 or pcDNA3.1-NC. (*P* < 0.01, Fig. 3b). Then, we further analyzed the mRNA expression level of E-cadherin in 72 paired CCRCC tissues and normal renal tissues (Supplementary Table S2). The results showed that the E-cadherin level was also significantly downregulated in the CCRCC tissues compared with the normal renal tissues (*P* < 0.01, Fig. 3c). Obviously, the expression levels of E-cadherin were related to clinical stage (Supplementary Table S2). Moreover, HOXD10 expression was positively correlated with E-cadherin in CCRCC tissues (*Rs* = 0.454, *P* < 0.01, Fig. 3c). Our data proved that E-cadherin is a potential target gene of HOXD10.

### HOXD10 regulated invasion and migration via the EMT process

**Fig. 4:**
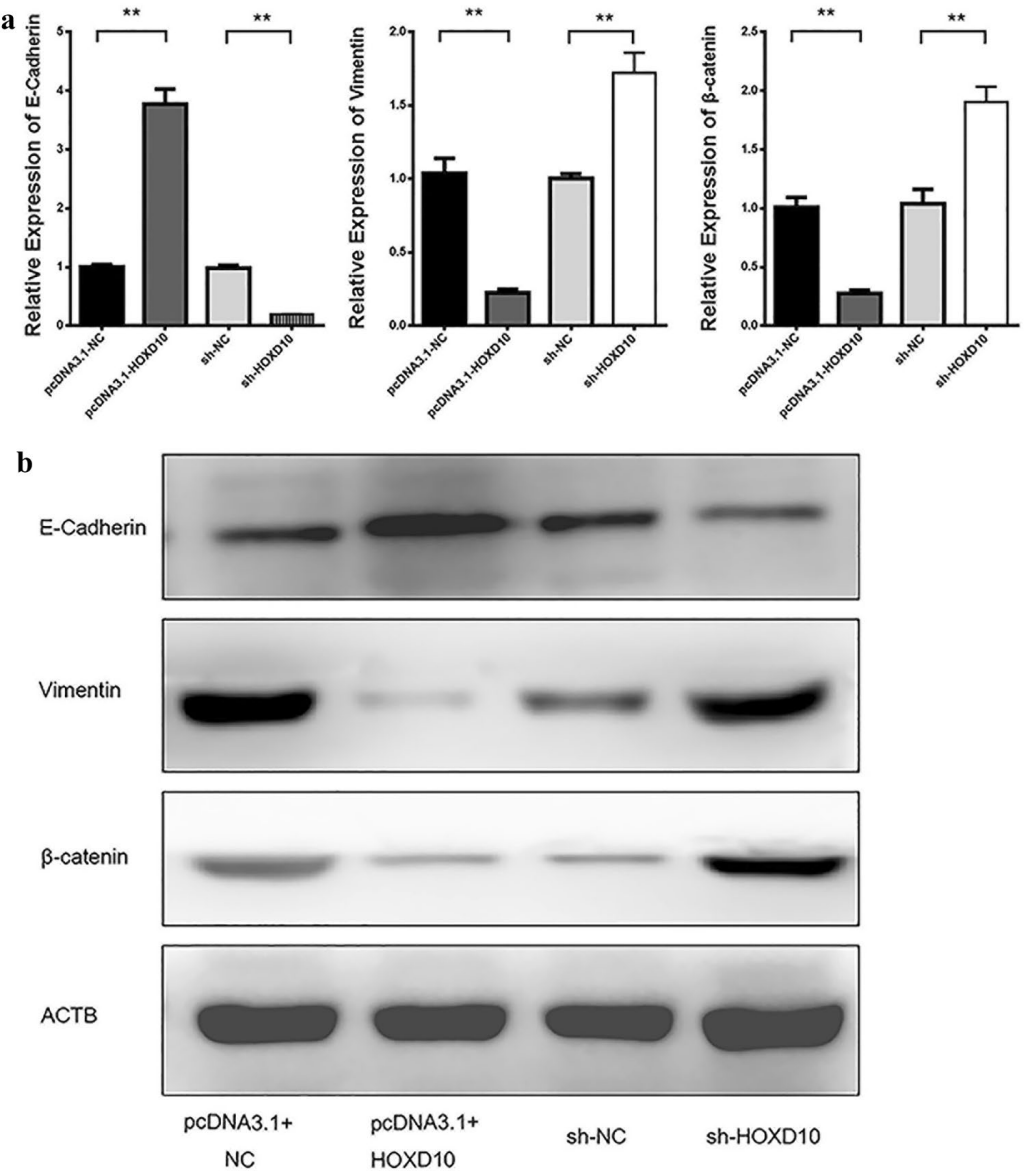
HOXD10 regulated the expression of E-cadherin and other EMT-related genes. **a** The mRNA expression levels of E-cadherin, vimentin, and β-catenin were examined by qRT-PCR after upregulation and knockdown of HOXD10 expression, **P < 0.01. **b** The protein levels of E‐cadherin, vimentin, and β-catenin were detected by Western blots, **P < 0.01

The mutation or deletion of E-cadherin, a well-known epithelial marker, is a pivotal process of EMT [13]. To further verify the molecular mechanism of HOXD10-mediated inhibition of invasion and migration of CCRCC cells, we detected the role of HOXD10 in EMT-associated genes. Upregulation of HOXD10 expression increased the level of E-cadherin and decreased the expression of vimentin and β-catenin (*P* < 0.01, Fig. 4a, b) at the mRNA and protein levels. In parallel, downregulation of HOXD10 expression led to a decline in E-cadherin levels and an increase in vimentin and β-catenin levels (*P* < 0.01, Fig. 4a, b). These data indicated that HOXD10 might inhibit the invasion and migration of RCC cells through the EMT process.

## Discussion

Homeobox (HOX) genes were originally discovered in *D. melanogaster* and encode a highly evolutionarily conserved subgroup of transcription factors [14]. In the human genome, the HOX family is organized into four clusters (HOXA, B, C, D), and HOX genes play crucial roles in regulating cell differentiation, angiogenesis, and receptor signaling pathways [15]. Previous studies have shown that aberrant HOX gene expression exists in both abnormal development and malignancy. First, a large proportion of HOX genes were thought to be oncogenes; however, subsequent research revealed that HOX genes also function in carcinoma suppression. As oncogenes, HOX genes are normally expressed during the embryonic period and are reactivated in carcinoma and show downregulated expression in normal differentiated adult tissues; in contrast, some HOX genes are expressed in normal differentiated adult tissues but show downregulated expression in carcinoma [4]. HOXD10 is a member of the HOX gene family and is abnormally expressed in several cancers, such as cholangiocellular cancer (CCC) and hepatocellular cancer [10, 16]. A previous study reported that HOXD10 expression was downregulated in cholangiocellular cancer and that upregulation of HOXD10 expression could decrease the invasiveness and migratory ability of CCC cells, revealing that HOXD10 played an inhibitory role in CCC progression [16]. Zhang et al. showed that the proliferative and invasive capabilities of HTR-8 cells were impaired in the pcDNA3.1-HOXD10 group but strengthened in the sh-HOXD10 group compared with the pcDNA3.1 group and sh-NC group, respectively [17]. However, the function and underlying mechanisms of HOXD10 in CCRCC have not been identified.

In the present study, we demonstrated that the mRNA and protein levels of HOXD10 were decreased in CCRCC tissues and RCC cell lines. The low expression level of HOXD10 in CCRCC tissues was closely related to the clinical stage of CCRCC patients, but the correlation between HOXD10 and histological grade was not significant. In parallel, to explore whether the expression of HOXD10 has any effect on RCC cell invasion, migration, proliferation, and colony formation in vitro, we carried out a series of biological function assays. In the translation of DNA into proteins, certain factors may hinder protein synthesis or cause extensive protein degradation, so, as shown in the result, the overexpression effect was up to 250 times in qRT-PCR while no similar effect was observed in WB. Upregulation of HOXD10 expression had a suppressive effect on the biological function of RCC cells; in contrast, knockdown of HOXD10 improved these abilities. Previous studies on ovarian cancer showed that downregulation of HOXD10 expression by miR-10b overexpression may enhance the migration and invasion in ovarian cancer cell lines by inducing an increase in prometastatic gene products [18]. Additionally, Li et al. studied the role of HOXD10 in the malignant biological behavior of the gastric cancer cell line SGC-7901. These researchers demonstrated that SGC‐7901 cell proliferation, migration, and invasion were significantly suppressed after successful transfection with pcDNA3.1‐HOXD10 compared with those of the pcDNA3.1 group [19]. Another pivotal study showed that the mRNA expression level of HOXD10 in stomach cancer tissues was remarkably lower than that in normal surrounding tissues, and the aberrant reduction of HOXD10 expression promoted migration and invasion in gastric cancer cells [20]. Based on this result, we preliminarily inferred that HOXD10 may serve as an antioncogene in CCRCC progression.

Previous studies have proven that the invasive and migratory ability of tumor cells is crucial to cancer metastasis [21]. Carrion et al. found that sustained expression of HOXD10 suppresses the expression of genes that directly affect remodeling of the extracellular matrix and cell migration during angiogenesis, such as α3-integrin and matrix metalloproteinase 14 (MMP-14) [22]. In addition, these genes have close links with invasion, migration, and tumor progression in a variety of carcinomas, such as breast tumors [23, 24]. These findings showed that HOXD10 functions as a general inhibitor of cell invasion. Epithelial-mesenchymal transition (EMT) is a process in which epithelial cells acquire mesenchymal features; in cancer, EMT is associated with tumor initiation, invasion, and metastasis [25]. Cancer cells lose the epithelial phenotype, which may contribute to invasion and metastasis through EMT, and they have the ability to cross endothelial barriers and enter the blood and lymphatic circulation [26]. The adhesion protein E-cadherin, as an epithelial marker, plays a central role in the process of epithelial morphogenesis and can prevent tumor metastasis by inhibiting EMT [27]. E-cadherin expression has been linked to the cellular functions of reduced invasiveness, growth inhibition, and differentiation [28]. A recent study reported that SIRT6 promoted invasion, migration, and EMT of HCC via the autophagic degradation of E-cadherin [29]. The genes regulated by the HOX gene include adhesion molecules, growth factors, and extracellular proteins. Therefore, we predicted that HOXD10 shared complementary binding sites with the 3′-UTR of E-cadherin by the online software PROMO. In the current study, we analyzed E-cadherin expression in the same 72 paired CCRCC tissues. We found that the level of E-cadherin was remarkably downregulated in CCRCC tissues and was positively correlated with the expression level of HOXD10. In addition, our study showed a significant correlation between downregulated E-cadherin expression and advanced clinical stage. Celebiler et al. found that underexpression of E-cadherin was significantly correlated with advanced stage breast cancer [30].

Moreover, ChIP and dual-luciferase reporter assays were applied to further verify that HOXD10 could bind to E-cadherin and regulate its expression. Our study demonstrated that HOXD10 could bind to the AACAAAAG region of the E-cadherin promoter and regulate its expression. The level of E-cadherin was remarkably decreased in the HOXD10 knockdown groups. In addition to epithelial markers, the induction of mesenchymal markers is an important phenotype in the EMT process [31, 32]. Therefore, we further detected the expression of EMT-related genes as the HOXD10 expression level changed. According to the qRT-PCR and western blot results, upregulation of HOXD10 expression could induce the expression of E-cadherin and decrease the expression of mesenchymal markers, such as vimentin. These findings are consistent with previous reports that HOXD10 inhibits ESCC cell invasion and migration by suppressing EMT [33]. The results revealed that HOXD10 might be an EMT-related transcription factor.

In addition, there are still several limitations in our study. First, the upstream regulatory mechanism leading to the decline of HOXD10 was not studied. Second, a larger study sample is needed to assess the relationship between HOXD10 and lymph node metastasis. Finally, the specific mechanism by which HOXD10 regulates E-cadherin expression and EMT remains unknown. Therefore, we will carry out a subsequent study to address these limitations.

In conclusion, we demonstrated that HOXD10 acts as a tumor suppressor in CCRCC. HOXD10 expression is downregulated in CCRCC tissues and RCC cells. HOXD10 may inhibit the invasion and migration of RCC cells by binding to E-cadherin and affecting the EMT process. Therefore, HOXD10 may serve as a potential therapeutic target for CCRCC patients.

## Supplementary Information

Below is the link to the electronic supplementary material.

Supplementary file1 (DOCX 16 kb)

Supplementary file2 (TIF 323 kb)

Supplementary file3 (DOCX 12 kb)

## Data Availability

The datasets analysed during the current study are available in the [StarBase-V3.0 database] repository, [http://starbase.sysu.edu.cn/panGeneDiffExp.php].
