# Supplementary material for: Upregulation of homeobox D10 expression suppresses invasion and migration of clear cell renal cell carcinoma through targeting of E-cadherin
Source: Mol Biol Rep. 2021 Nov 25;49(3):1837–46. doi: 10.1007/s11033-021-06993-8 (PMC8863706; doi:10.1007/s11033-021-06993-8)
Supplement: Supplementary file 1 — Supplementary file1 (DOCX 16 kb) [file 11033_2021_6993_MOESM1_ESM.docx]

**Supplementary Table S1**. Primers Used for qRT-PCR Analysis.

| target genes | primers | Product length (bp) | Tm (℃) |
| --- | --- | --- | --- |
| HOXD10 | F: TGAGGTCTCCGTGTCCAGTC | 182 | 60 |
|  | R: CGCGGGTGAGGTACATATTG |  |  |
| E-cadherin | F: CGAGAGCTACACGTTCACGG | 162 | 60 |
|  | R: GGCCTTTTGACTGTAATCACACC |  |  |
| Vimentin | F: CGCCTGCAGGATGAGATTCAG | 175 | 60 |
|  | R: TCAGGGAGGAAAAGTTTGGAAGA |  |  |
| β-catenin | F: GGCTACTGTTGGATTGATTC | 161 | 60 |
|  | R: CCACAAATTGCTGCTGTGTC |  |  |
| GAPDH | F: AGGGGTCATTGATGGCAACA | 104 | 60 |
|  | R: AGGGGTCATTGATGGCAACA |  |  |

**Supplementary Table S2.** E-cadherin mRNA levels in relation to clinical-pathological

parameters of CCRCC patients

| Group | N | Mean±SD | t | *P-Value* |
| --- | --- | --- | --- | --- |
| Age (years) |  |  |  |  |
| ≤60 | 49 | 0.430+0.875 | 0.736 | 0.464 |
| >60 | 23 | 0.292+0.293 |  |  |
| Gender |  |  |  |  |
| Male | 49 | 0.414+0.874 | 0.457 | 0.649 |
| Female | 23 | 0.328+0.318 |  |  |
| Lymph node metastasis | | | | |
| Negative | 66 | 0.414+0.768 | 1.070 | 0.288 |
| Positive | 6 | 0.077+0.056 |  |  |
| Histological grade | | | | |
| Well/moderate | 65 | 0.413+0.774 | 0.897 | 0.373 |
| Poor | 7 | 0.147+0.175 |  |  |
| TNM stage | | | | |
| Ⅰ | 49 | 0.436+0.486 | 3.180 | 0.029 |
| Ⅱ | 8 | 0.330+0.278 |  |  |
| Ⅲ | 6 | 0.057+0.027 |  |  |
| Ⅳ | 9 | 0.053+0.031 |  |  |
