# Supplementary material for: Upregulation of homeobox D10 expression suppresses invasion and migration of clear cell renal cell carcinoma through targeting of E-cadherin
Source: Mol Biol Rep. 2021 Nov 25;49(3):1837–46. doi: 10.1007/s11033-021-06993-8 (PMC8863706; doi:10.1007/s11033-021-06993-8)
Supplement: Supplementary file 3 — Supplementary file3 (DOCX 12 kb) [file 11033_2021_6993_MOESM3_ESM.docx]

**Figure Captions**

Supplementary Fig. S1 StarBase-V3.0 database demonstrates that the expression level of HOXD10 is downregulated in KIRC tissues compared with the corresponding normal renal tissues. (KIRC also named as clear cell renal cell carcinoma)
